# New Theoretical Model to Describe Carrier Multiplication in Semiconductors: Explanation of Disparate Efficiency in MoTe_2_ versus PbS and PbSe

**DOI:** 10.1021/acs.jpcc.4c00383

**Published:** 2024-02-28

**Authors:** Sven Weerdenburg, Nisha Singh, Marco van der Laan, Sachin Kinge, Peter Schall, Laurens D. A. Siebbeles

**Affiliations:** † Chemical Engineering Department, Delft University of Technology, Van der Maasweg 9, Delft 2629 HZ, The Netherlands; ‡ Materials Research & Development, Toyota Motor Europe, Zaventem B1930, Belgium; § Institute of Physics, University of Amsterdam, Amsterdam 1098 XH, The Netherlands

## Abstract

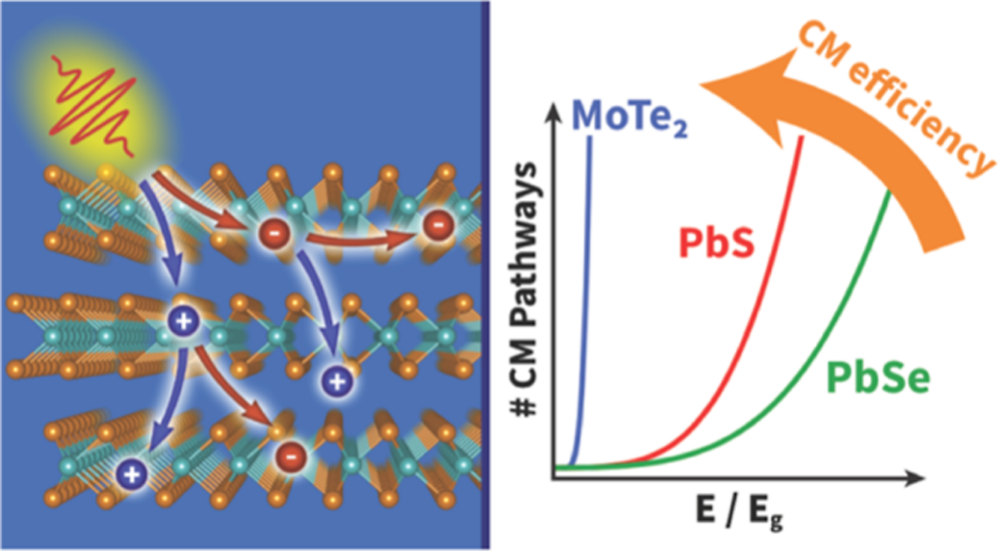

We present a theoretical model to compute the efficiency of the generation of two or more electron–hole pairs in a semiconductor by the absorption of one photon via the process of carrier multiplication (CM). The photogeneration quantum yield of electron–hole pairs is calculated from the number of possible CM decay pathways of the electron and the hole. We apply our model to investigate the underlying cause of the high efficiency of CM in bulk 2H–MoTe_2_, as compared to bulk PbS and PbSe. Electronic band structures were calculated with density functional theory, from which the number of possible CM decay pathways was calculated for all initial electron and hole states that can be produced at a given photon energy. The variation of the number of CM pathways with photon energy reflects the dependence of experimental CM quantum yields on the photon energy and material composition. We quantitatively reproduce experimental CM quantum yields for MoTe_2_, PbS, and PbSe from the calculated number of CM pathways and one adjustable fit parameter. This parameter is related to the ratio of Coulomb coupling matrix elements and the cooling rate of the electrons and holes. Large variations of this fit parameter result in small changes in the modeled quantum yield for MoTe_2_, which confirms that its high CM efficiency can be mainly attributed to its extraordinary large number of CM pathways. The methodology of this work can be applied to analyze or predict the CM efficiency of other materials.

## Introduction

The development of a new generation solar cells requires exploring ways to surpass the Shockley–Queisser limit of 33.7%.^1,2^ One of these ways is to enhance the photocurrent of a solar cell via excitation of two or more electrons by the absorption of a single photon.^3^ This process of carrier multiplication (CM) can raise the power conversion limit of a single-junction solar cell to 46%.^3,4^ Apart from solar cells, CM is also of interest to the development of more efficient photodiodes since it enhances the photocurrent. Note that CM has also been referred to as electron–hole pair multiplication (EHPM) or impaction ionization (II) in bulk semiconductors and has been called multiexciton generation (MEG) in quantum confined systems where excitons are formed rather than free charge carriers.^3,5^

**Figure 1:**
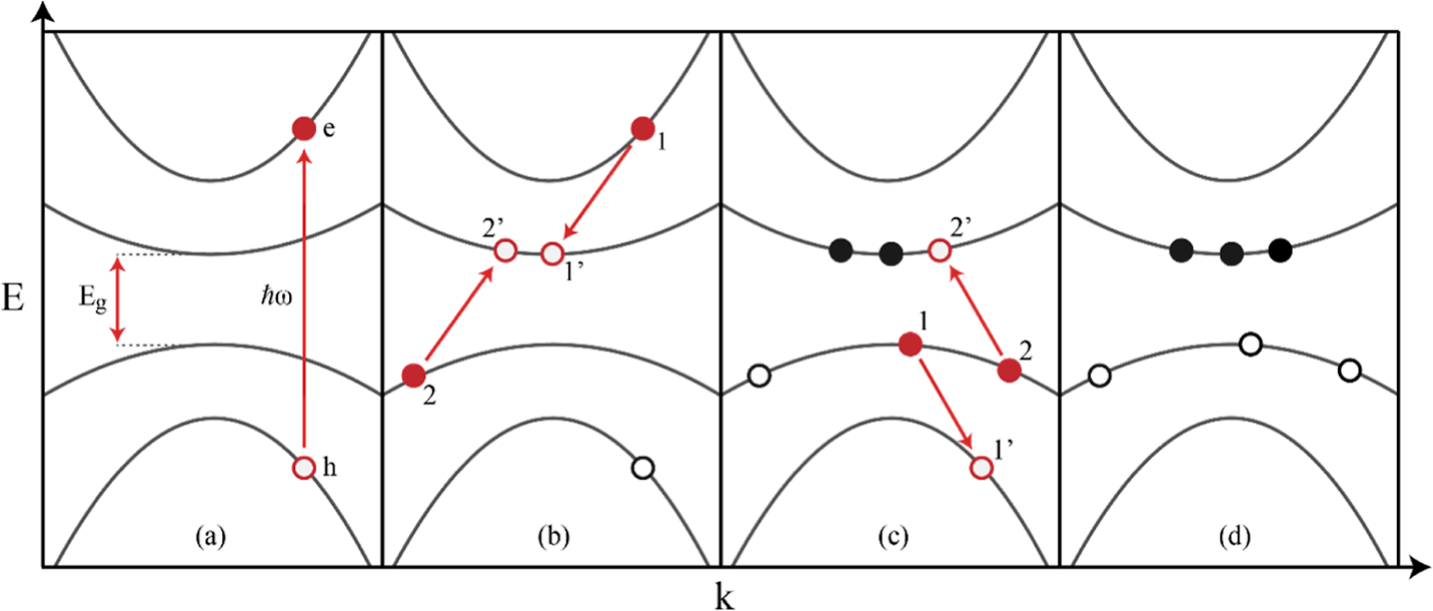
CM is illustrated in a band structure diagram. (a) Photoexcitation of a valence band electron with energy *ℏω* leads to the generation of an electron–hole pair. In this example, both photogenerated carriers can scatter to yield multiple electron–hole pairs, which is illustrated as a subsequent processes in the following subpanels. (b) The photogenerated electron in state 1 transfers energy to a valence band electron in state 2, resulting in the two final states 1′ and 2′ to be occupied by the electrons. (c) Scattering between two valence band electrons initially in states 1 and 2 results in the relaxation of the electron from state 1 to a lower empty valence band state 1′, this is equivalent to the relaxation of a hole from state 1′ to state 1. The simultaneous excitation of an electron from state 2 to conduction band state 2′ ensures that energy is conserved. (d) The two scattering processes in (b) and (c) finally yield three electron–hole pairs and thus a quantum yield of 3.

A CM process is schematically shown in Figure 1a. It starts with photoexcitation of a single electron across the band gap of a semiconductor. If the photoexcited electron has sufficient excess energy, exceeding that of the band gap (*E*_g_), it can undergo Coulomb scattering with a valence electron and excite the latter to the conduction band. As depicted in Figure 1b, this leads to the formation of an additional electron–hole pair. Note that the initially photogenerated hole in the valence band can also relax by CM provided its energy is at least 1*E*_g_ below the top of the valence band, as shown in Figure 1c. Since either the electron or the hole must have an excess energy above *E*_g_, CM is possible for photons with energy above twice the band gap, i.e., *ℏ*ω ≥ 2*E*_g_. In the most ideal case, CM thus has an onset photon energy of 2*E*_g_ and the number of electron–hole pairs produced per absorbed photon increases with one for each further photon energy increment of *E*_g_.^3^ The number of electron–hole pairs produced per absorbed photon, which is known as the electron–hole pair photogeneration quantum yield, then exhibits a steplike dependence on the photon energy. However, photons with energy equal to a multiple of the band gap will, in general, not transfer their entire excess energy to either an electron or a hole. In addition, CM occurs in competition with energetic relaxation of electrons and holes by cooling via phonon emission, so that their initial excess energy above the band edges is not fully available for CM. These factors cause the onset energy of CM to be usually larger than twice the band gap and the dependence of the CM quantum yield on photon energy to deviate from the ideal steplike behavior.

CM has been studied for a variety of materials, most notably Pb-chalcogenides,^6−9^ Cd-chalcogenides,^10−12^ Si nanostructures,^13,14^ percolative networks,^4,15^ perovskites,^16−19^ and transition-metal dichalcogenides (TMDC).^20−22^ Particularly, 2H-TMDCs, where the prefix 2H- denotes a trigonal prismatic phase structure, have gained much attention due to reports of a remarkably high efficiency of bulk CM,^20−22^ which highly exceeds that of bulk PbS and PbSe.^7^ For the 2H–MoTe_2_ and 2H–WSe_2_ TMDCs, the onset energy of CM was found to be close to a photon energy of 2*E*_g_ and the quantum yield of electron–hole pairs exhibits an almost ideal steplike dependence on photon energy.^20,21^ This is very different for bulk PbS and PbSe where the onset energy of CM is as high as ∼6*E*_g_ and the increase of the CM quantum yield with photon energy is much smaller.^7^ The origin of the highly efficient CM in these TMDCs has not been rationalized before. A high CM quantum yield requires the rate *R*_CM_ of CM to be (much) larger than the rate *R*_cool_ of charge carrier cooling, i.e., their ratio (*R*_CM_/*R*_cool_) to be large. In the studies on TMDCs mentioned above, the CM rate was estimated to be an order of magnitude larger than the cooling rate.^20,21^ A recent theoretical study on TMDCs found that electron–phonon coupling could reduce the band gap upon photoexcitation, which could enable a CM onset even below 2*E*_g_ and thereby increase the CM efficiency.^23^ Furthermore, weak carrier-phonon coupling^24^ and long-lived optical phonon modes^25^ have been observed in 2H–MoTe_2_ that could contribute to a low carrier cooling rate. While these are important findings, they do not directly explain why CM in TMDCs is much more efficient than in other bulk materials such as PbS and PbSe.

In this paper, we present a theoretical model that derives the CM efficiency from the number of CM pathways. We apply this model to explain why the experimental CM efficiency of MoTe_2_^20,21^ is much higher than measured for PbS and PbSe.^7^ From band structures obtained with density functional theory (DFT), we evaluate all possible Coulomb scattering pathways of photogenerated electrons and holes that lead to the generation of an additional electron–hole pair. The experimental disparate CM quantum yields of MoTe_2_ versus PbS and PbSe can be described qualitatively based on the very different numbers of CM decay pathways of initially energetic electrons and holes produced by photoexcitation. We quantitatively reproduce the experimental CM quantum yields as a function of photon energy with only one adjustable parameter for each material, which relates to the ratio of the Coulomb interaction strength involved in CM and the charge carrier cooling rate. Our model provides an explanation for the high CM efficiency in MoTe_2_, as opposed to the much lower CM efficiencies in PbS and PbSe. In our model the Coulomb interaction strength, cooling rate, and optical oscillator strength for photoexcitation were taken to be independent of the electronic states involved. This leads to the conclusion that the variation of the CM rate with photon energy is mainly determined by the number of CM pathways for the decay of electrons and holes. The theoretical framework of this study can be applied to investigate CM efficiencies for other (unexplored) materials.

## Theoretical Methods

### Electronic Band Structure Calculations

Electronic band structures of MoTe_2_, PbS, and PbSe were obtained from DFT calculations with the ABINIT package.^26^ All calculations were done using optimized norm-conserving Vanderbilt pseudopotentials,^27^ a generalized gradient approximation (GGA) exchange–correlation functional,^28^ and included spin–orbit coupling. The geometries were based on lattice constants reported literature and are listed in Table S1 in the Supporting Information.^29,30^ We used a *k*-point grid of *n*_k_ = 14 × 14 × 7 crystal momenta for MoTe_2_ and *n*_k_ = 16 × 16 × 16 for PbS and PbS. The convergence condition between two self-consistent field steps was chosen to be a 10^–8^ hartree (Ha) absolute difference in total energy. Cutoff energies were chosen to be 30, 25, and 40 Ha for MoTe_2_, PbS, and PbSe, respectively. Band structure diagrams were obtained from *k*-points along the symmetry lines of the irreducible Brillouin zone, whereas the density of states and the number of CM pathways were computed from *k*-points in the full Brillouin zone. The conduction bands were shifted using a scissor operator to match the experimentally determined band gaps reported in the literature, which is further discussed below.

### Calculation of the Number of Carrier Multiplication Pathways

The number of CM pathways can be obtained by forming sets of charge carrier states and counting each set that fulfills the conditions for an allowed CM pathway. As is shown in Figure 1b, a CM event involves four electronic Bloch states. A primary electron in the initial state with index 1 and energy *E*_1_ decays to a final state 1′ at energy *E*_1′_. This decay is accompanied by exciting a secondary electron from state 2 with energy *E*_2_ in the valence band to state 2′ with energy *E*_2′_ in the conduction band. Conservation of energy applies such that Δ*E*_1′,2′,1,2_ = *E*_1′_ + *E*_2′_ - *E*_1_ - *E*_2_ = 0. Apart from conservation of energy, Coulomb scattering requires the difference of the initial and final crystal momenta ***k***_*i*_ of the electrons to be a reciprocal lattice vector ***G***, which is zero for Normal-type scattering and nonzero for Umklapp-type scattering.^31,32^ The conservation condition of crystal momentum is thus
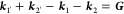
1

Furthermore, in the case an electron initiates CM (Figure 1b), the condition *E*_CBM_ ≤ *E*_1′_ < *E*_1_ applies for the primary electron in state 1, where *E*_CBM_ denotes the minimum energy of the lowest conduction band and *E*_1_ (*E*_1′_) is the energy of the electron in its initial (final) state. As shown in Figure 1c, CM by relaxation of a hole involves scattering between two valence electrons, where the primary electron 1 remains in the valence band, and thus, the condition *E*_VBM_ ≥ *E*_1_ > *E*_1′_ must be fulfilled, where *E*_VBM_ denotes the maximum valence band energy. Finally, the secondary electron in valence band state 2 must be excited to a conduction band state 2′, i.e., *E*_2_ ≤ *E*_VBM_ < *E*_2′_, which then along with the aforementioned conditions also implies that the energy loss of the primary electron is greater than or equal to the band gap, *E*_1_ – *E*_1′_ ≥ *E*_g_.

The conservation of energy and crystal momentum and the conditions for the initial and final states are used to compute the number of CM pathways. The discrete electronic Bloch states obtained from DFT calculations are characterized by a wave vector ***k***_*i*_ in the first Brillouin zone, a band index ν_*i*_ and an energy *E*_*i*_ (ν_*i*_, ***k***_*i*_). By making all possible combinations of the initial and final states of primary and secondary electronic states and assessing the aforementioned conditions, the number of possible CM pathways for any initial primary carrier state can be counted. This leads to the following expression of the number of CM pathways for the initial primary carrier state 1
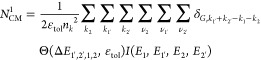
2where
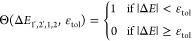
3and
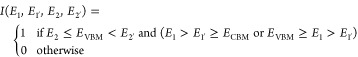
4

In the summation over initial and final states, the conservation of crystal momentum is taken into account by a Kronecker delta δ_***G***, ***k***1_′_ + ***k***2_′_ – ***k***1 – ***k***2_. This condition can be satisfied numerically since the DFT calculations provide electronic states on a regular grid of *k*-points. Conservation of energy of the discrete electronic Bloch states leads to the requirement Δ*E*_1′,2′,1,2_ = 0. However, this constraint is too strict since the DFT calculations provide energies on a grid of a finite number of *k*-points. In addition, consideration of electronic energy only is insufficient since CM can be assisted by absorption or emission of phonons. Therefore, the energy conservation rule is relaxed by using a top-hat function Θ(Δ*E*, ε_tol_) as defined in eq 3, analogous to previous studies.^33,34^ The parameter ε_tol_ relaxes energy conservation over the tolerance interval 2ε_tol_ and the function Θ(Δ*E*, ε_tol_)/2ε_tol_ can be considered as a broadened energy conserving Dirac delta function δ(Δ*E*). The function *I*(*E*_1_, *E*_1′_, *E*_2_, *E*_2′_) defined in eq 4 checks the energy requirements for the initial and final states of the primary and secondary carriers and therefore distinguishes a CM pathway from any ordinary scattering pathway. The summation in eq 2 over all final states at the *k*-points (***k***_1′_, ***k***_2′_) scales in a trivial manner with the square of the number of *k*-points (*n*_*k*_) included in the DFT calculations. Note that the fourth *k*-point (***k***_2_) is fixed due to conservation of crystal momentum and therefore does not contribute to this scaling factor. Therefore, we divide the summation by *n*_*k*_^2^, so that for sufficiently large *n*_*k*_ the value of *N*_CM_^1^ converges to a constant value and results for different sizes of *k*-point grids can be directly compared. Hence, for each combination of band indices υ_*i*_ in eq 2 the value of *N*_CM_^1^ represents the fraction of all *n*_*k*_^2^ final states 1′ and 2′ per unit energy that can be reached by CM decay of an electron from the initial state with index 1, which excites another electron from the state with index 2. The unit of *N*_CM_^1^ is further discussed in the Supporting Information.

Values of *N*_CM_^1^ were calculated for all initial hole states in the valence bands and electron states in the conduction bands obtained from DFT calculations. In determining the states involved in absorption of a photon with energy ℏω the photon momentum was neglected, so that Δ***k*** = 0 for an optical transition. A constant optical oscillator strength was assumed for all optical transitions. Below, we refer to *N*_CM_^1^ as the density of the CM pathways.

### Modeling the Quantum Yield from the Density of Carrier Multiplication Pathways

Below, we describe our model to obtain the CM quantum yield from the density of CM pathways given by *N*_CM_^1^ in eq 2. The CM quantum yield is defined as the ratio between the number of generated electron–hole pairs and the number of absorbed photons, i.e.
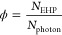
5

If an incident photon carries at least two band gap multiples of energy (*ℏ*ω ≥ 2*E*_g_), it becomes energetically possible that the photogenerated electron or hole induces CM to yield a second electron–hole pair. At a photon energy of three band gap multiples or more (*ℏ*ω ≥ 2*E*_g_), a primary carrier may have an excess energy above ≥2*E*_g_, so that more CM steps are possible and three or more electron–hole pairs can be produced. Due to computational limitations, these subsequent CM events are not taken into account. Therefore, for photon absorption event *i*, the quantum yield is determined by the probabilities of CM for the initially photogenerated electron (*P*_CM_^e_*i*_^) and hole (*P*_CM_^h_*i*_^) only, and can have a value of 1 up to 3, i.e.
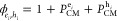
6where e_*i*_ and h_*i*_, respectively, denote the conduction and valence band states occupied by the photogenerated primary carriers (electron and hole).

We assume that directly after photoexcitation a generated carrier can follow only one of two paths: (1) it induces CM by Coulomb scattering and yields an additional electron–hole pair or (2) it relaxes by phonon emission without generating an additional electron–hole pair. Carrier cooling is thus modeled as a single step, and a possible sequence of cooling and CM steps is neglected. From these assumptions, the probability of CM for carrier *i* is then defined as the ratio between the rate of CM and the sum of the CM and carrier cooling rates
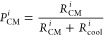
7where *R*_CM_^*i*^ and *R*_cool_^*i*^ are the total CM and carrier cooling rates, respectively, for a primary carrier in initial state *i*.

CM rates have been calculated using ab initio and semiempirical methods for several bulk semiconductors and quantum dots.^7,35−47^ In these methods, Fermi’s Golden Rule is used, which requires Coulomb matrix elements of all initial and final states. Fortunately, it has been found earlier that the Coulomb matrix elements do not depend much on the electronic states involved in different CM pathways.^38,39,43,44,46−50^ Hence, to a good approximation, the Coulomb matrix elements can be kept at a constant value, thereby assuming that each single scattering event occurs at an equal rate. As described in the Supporting Information, the total CM rate for primary carrier in initial state *i* is then directly proportional to the density of CM decay pathways, *N*_CM_^*i*^, in eq 2, according to
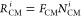
8with the prefactor *F*_CM_ taking into account Coulomb coupling, which is assumed to be independent of the initial and final states. Using eq 8 for the CM rate, the probability of CM in eq 7 can be rearranged to
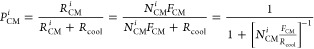
9

This equation describes the probability of CM for a carrier in the initial state *i*. The quantum yield for photon absorption event *i*, that generates the primary initial electron state e_i_ and hole state h_i_ is obtained by substitution of eq 9 for the CM probabilities into eq 7, yielding
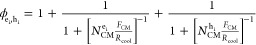
10

A photon with energy *ℏ*ω can induce different optical transitions from the valence band to conduction band and thereby can result in the generation of different initial states of a pair of an electron and a hole, e_*i*_ and h_*i*_, with different values for *N*_CM_^e_i_^ and *N*_*CM*_^h_*i*_^. Assuming a constant optical oscillator strength, the quantum yield at photoexcitation with energy in a small range between ℏω and ℏω + Δℏω is the average quantum yield, Φ(ℏω), of the total number of possible optical photoexcitations, *N*_ℏω_, so that
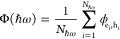
11

Finally, substituting eq 10 into eq 11 results in the CM quantum yield
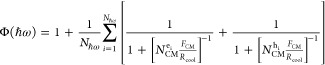
12

As discussed below, we calculate *N*_CM_ using band structures obtained from DFT and fit eq 12 to experimental quantum yields with *F*_CM_/*R*_cool_ as an adjustable parameter.

The equations above are valid for the physically realistic case of a nonzero cooling rate. However, to obtain insights into the effect of cooling, we also consider the CM quantum yield that would be obtained in absence of cooling, i.e., *R*_cool_ →0. In that case, the quantum yield is given by
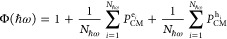
13with *P*_CM_^*e*_*i*_^ = 1 if *N*_CM_^*e*_i_^ > 0, so that CM is possible, and *P*_CM_^*e*_*i*_^ = 0 if *N*_CM_^*e*_*i*_^ = 0, and analogously for the hole. Hence, the second and third terms in eq 13 are the fractions of the initially photoexcited electrons and holes that decay by the CM, respectively.

## Results and Discussion

### Calculated Band Structures and Density of States

**Figure 2:**
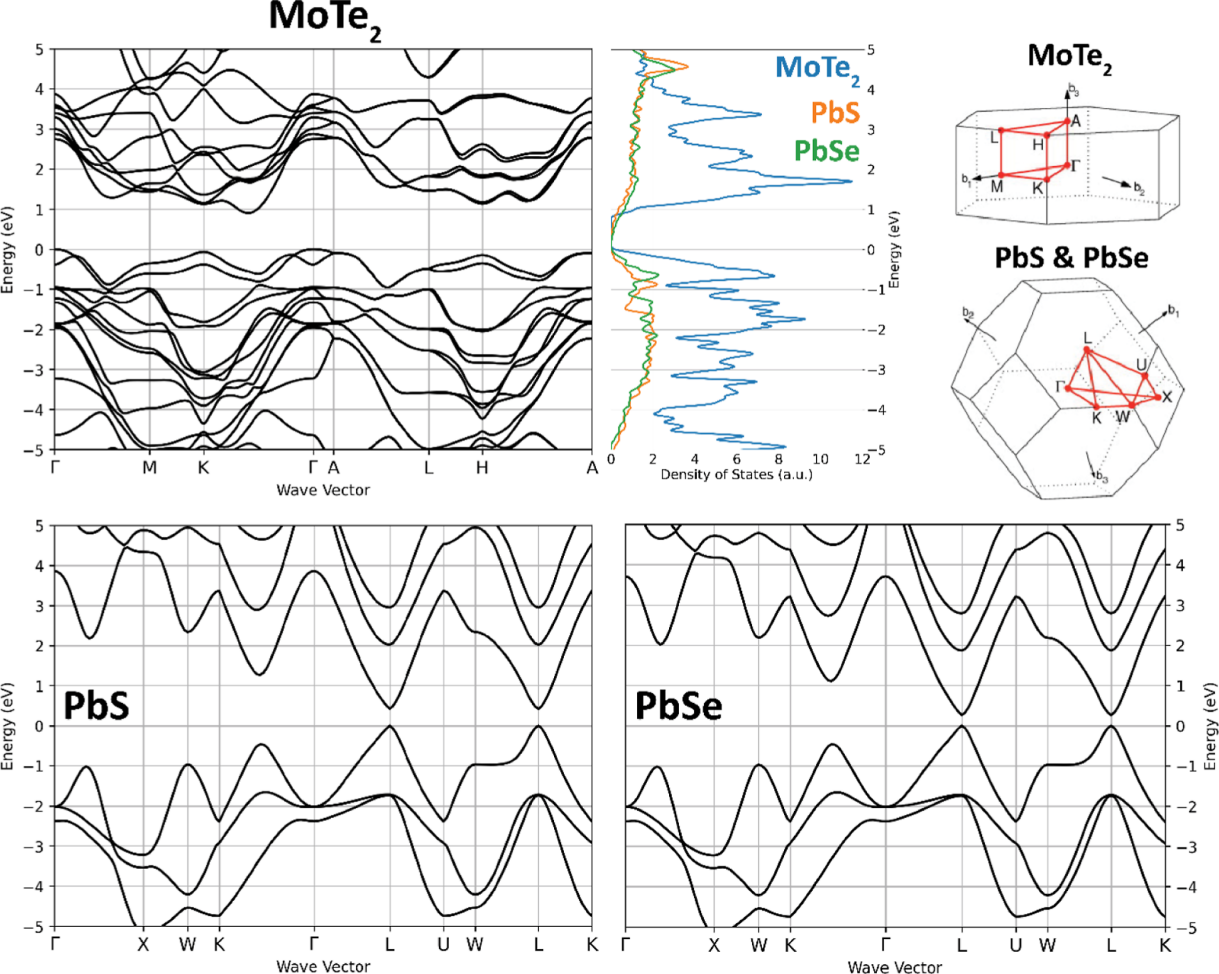
Band structures, density of states, and the Brillouin Zones of 2H–MoTe_2_, PbS, and PbSe. Brillouin Zone illustrations taken with permission from ref (52). Licensed under a Creative Commons Attribution License.

The band structures and density of states obtained from the DFT calculations are shown in Figure 2. The calculated band gaps for MoTe_2_, PbS, and PbSe are 0.72, 0.30, and 0.32 eV, respectively. A scissor operator was applied to match the experimental band gaps of 0.85 eV for MoTe_2_^20^ and 0.42 and 0.26 eV for PbS^51^ and PbSe,^51^ respectively. It should be noted that Zheng et al. and Kim et al. reported slightly different band gaps for MoTe_2_, where Kim et al. found an indirect band gap of 0.85 eV and Zheng et al. an excitonic band gap of 0.9 eV.^20,21^ This difference may be due to different sample thicknesses, which were 16.4 and 5 nm for Kim et al. and Zheng et al., respectively. Since our calculations were done on bulk 2H–MoTe_2_, the band gap reported by Kim et al. was used.

From the band structures, it is clear that the bands of MoTe_2_ are relatively flat compared to the more curved bands of the lead chalcogenides. Moreover, MoTe_2_ has a larger number of bands within the shown energy range, which results in a density of states that is larger than that of the lead chalcogenides. It can also be seen that many of the electronic bands of MoTe_2_ appear as “bundles”, where multiple bands overlap or lie closely together at many values of *k*. The relatively flat and bundled bands of MoTe_2_ result in large peaks in the density of states. For PbS and PbSe, at a low photon energy, the excitation occurs around the *L*-point. Due to the similar curvature of the valence and conduction bands near the *L*-point, the excess photon energy is divided almost equally over the hole and the electron. As a result, the energy onset of CM is much higher than 2*E*_g_.

### Calculated Density of CM Pathways and Experimental Quantum Yields

The density of CM pathways was computed from eq 2 with energies of the valence and conduction band states from DFT calculations covering the full Brillouin zone. All electronic states that can be generated by photons with energy up to 4 eV were included. Energy conservation was ensured within a range of ε_tol_ = 30 meV in eq 2, which is suitable for the convergence of the results, see the left panel of Figure S1.

**Figure 3:**
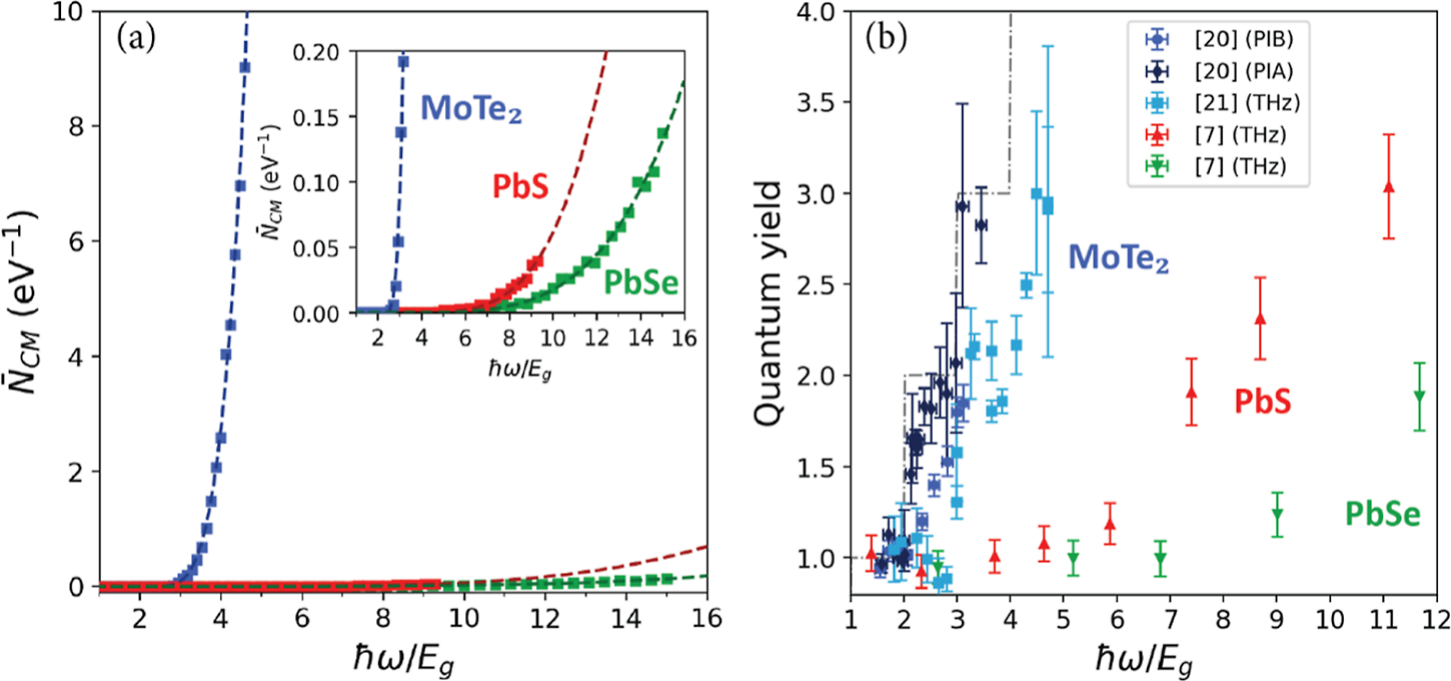
(a) Computed average density of CM pathways, , in energy intervals of intervals of Δ*ℏ*ω = 0.1 eV for MoTe_2_, PbS, and PbSe, as a function of band gap multiple. The dashed lines represent the best fit of eq 14 with values of *A* and *P* in Table 1. (b) The experimental quantum yield for 2H–MoTe_2,_^20,21^ PbS,^7^ and PbSe^7^ as a function of band gap multiple.

Figure 3a shows the summed average density of CM pathways, , as a function of the band gap multiple of the photon energy (*ℏ*ω/*E*_g_). In literature, the rate of impact ionization has been described analytically in the form of *R* = *A*(*E* – *E*_0_)^P^.^37,39,40,53^ We fit the average density of CM pathways similarly, according to
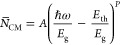
14where *A* and *P* are fitting parameters, and *E*_th_ is the lowest energy for which we find that *N*_cm_ > 0. The dashed lines in Figure 3a are the results of fits of eq 14 to *N̅*_CM_. *E*_th_ and the optimal values of *A* and *P* are given in Table 1. The fit reproduces the data for *N̅*_CM_ very well. It is clear from Figure 3a and the values of *A* and *P* that *N̅*_CM_ increases much more rapidly with photon energy for MoTe_2_ than for the lead chalcogenides. Figure 3b shows experimental quantum yields obtained from photoinduced bleaching (PIB), intraband absorption (PIA), or terahertz (THz) conductivity measurements.^20,21^ Comparing Figure 3a,b shows that the calculated onset of the rise of *N̅*_CM_ agrees very well with the experimental onsets of CM, which are close to 2.8*E*_g_ for MoTe_2_, 5.5*E*_g_ for PbS, and 7.0*E*_g_ for PbSe. The calculated trend of the slopes of *N̅*_CM_ in Figure 3a reflects that of the experimental CM quantum yields in Figure 3b, with the slope drastically decreasing in the order MoTe_2_, PbS, and PbSe. Hence, the much higher experimental CM quantum yield for MoTe_2_ can be explained qualitatively by the higher density of CM pathways than for PbS and PbSe.

**Table 1:** Values of *A* and *B* from Fit of eq 14 to the *N̅*_CM_ Data in Figure 3a

material	*A* (eV^–1^)	*P*	*E*_th_ (eV)	*E*_th_/*E*_g_
2H–MoTe_2_	0.26	4.1	1.9	2.2
PbS	4.6 × 10^–5^	3.8	1.4	3.3
PbSe	7.6 × 10^–6^	3.9	0.7	2.7

### Quantitative Modeling of Experimental Quantum Yields

We used our model outlined above to get insights into the origin of the much higher experimental CM quantum yields reported for MoTe_2_,^20,21^ as compared to PbS and PbSe.^7^ We fit eq 12 to the experimental quantum yields with *F*_CM_/*R*_cool_ as the only adjustable parameter. To get insights into the effect of cooling, we also calculated the upper limit of the quantum yield that is reached in the absence of cooling according to eq 13. In the latter case, the quantum yield is solely determined by the presence or absence of at least one CM pathway for each initial state of a carrier.

**Figure 4:**
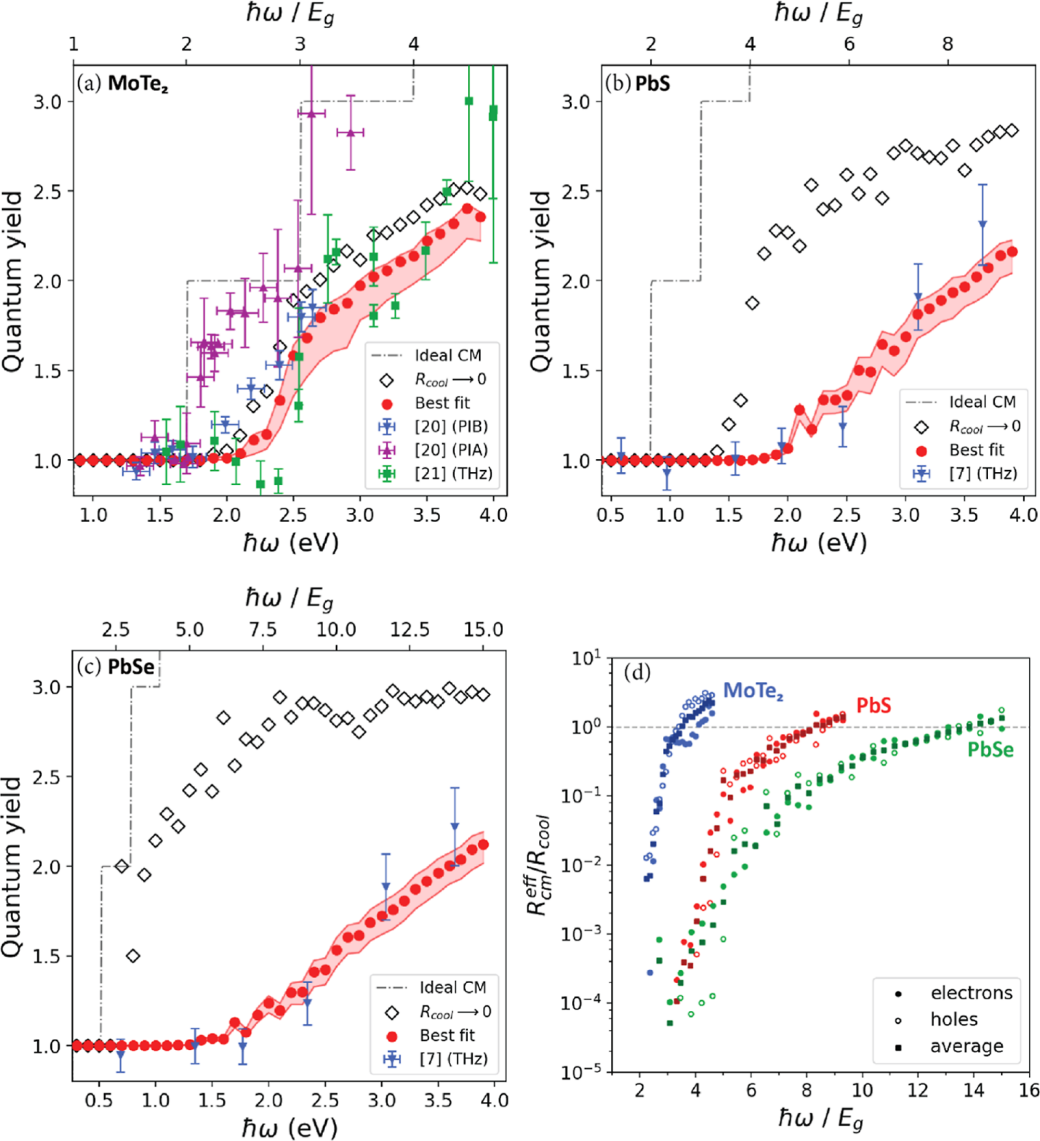
Quantum yields from experiment, best fits of eq 12, and calculated for *R*_cool_ → 0 according to eq 13 for (a) MoTe_2_, (b) PbS, and (c) PbSe. The shaded areas represent the one standard deviation interval of the fit, obtained from the variance of the parameter estimate (see SciPy documentation scipy.optimize.curve_fit). The dashed step-like curves illustrate the case of ideal CM. (d) The effective ratio of CM and carrier cooling rates for electrons, holes, and the average of both carriers obtained from eq 15 with the results from fitting eq 12 to the experimental quantum yield. Note that the fluctuations in the calculated quantum yield will in part be due to variations in *N*_CM_ due to the use of a finite *k*-point grid, see right panel of Figure S1. This effect is largest at low photon energies, at which only a few optical transitions are possible.

The experimental CM quantum yields for MoTe_2_ obtained from transient absorption spectroscopy (PIB and PIA) by Kim et al.^20^ and THz conductivity measurements by Zheng et al.^21^ are shown in Figure 4a. The quantum yields obtained from PIB are similar to those from the THz conductivity measurements, while PIA gives higher CM quantum yields. The discrepancy between the yields obtained from PIB and THz measurements and those from PIA could be due to a smaller signal-to-noise ratio for the latter, as reflected in the larger error bars for the PIA data. The larger effect of noise for PIA may stem from the fact that the change in transmission during PIA experiments was about 1 order of magnitude smaller than for PIB.^20^ The experimental CM quantum yields for PbS and PbSe in Figure 4b,c have been obtained from THz conductivity measurements by Pijpers et al.^7^

The results of fitting eq 12 to experimental quantum yields are shown in Figure 4a,c. The best fit of our model to the experimental data is shown as red dots and the one standard deviation interval as the shaded area. The open diamonds in Figure 4a–c represent the quantum yield calculated in the absence of cooling (*R*_cool_ → 0) according to eq 13. Figure 4a shows the best fit of our model to the experimental PIB and THz data for MoTe_2_.^20,21^ The fit agrees well with the measured results. Much like the THz data^21^ the modeled quantum yield of MoTe_2_ exhibits a step-like increase near 2.5 eV. The higher CM quantum yields obtained from PIA measurements cannot be reproduced with our model, even in case the maximum quantum yield of CM in the absence of cooling (*R*_cool_ → 0) is calculated. We therefore did not include the PIA data in our best fit and fitted our model only to the PIB and THz data. As mentioned above, this may be (in part) due to an uncertainty of the PIA data, and it will be of interest to repeat PIA and PIB experiments on MoTe_2_. Figure 4b,c shows that our model also reproduces the CM quantum yields for PbS and PbSe very well.

Note that in the absence of cooling (*R*_cool_ → 0) we do not obtain the dashed step-like increase of the quantum yield with photon energy, which implies that only part of all initially photogenerated electrons and holes can decay by CM. This is due to the restrictions of conservation of crystal momentum and energy. Interestingly for MoTe_2_ the quantum yield without cooling is not much larger than the results from the fit. This means that CM is much faster than cooling in MoTe_2_, in agreement with the conclusion by Zheng et al. that the rate for the first CM step is nearly an order of magnitude larger than the cooling rate.^21^ By contrast, in the absence of cooling, the quantum yields of PbS and PbSe are much higher than those obtained from the best fits; see Figure 4b,c. The latter implies that cooling significantly competes with CM in PbS and PbSe. Figure 2 shows that the electronic bands of MoTe_2_ are relatively flat compared to the more curved bands of PbS and PbSe. In the case of more flat bands, a change of the *k*-vector of an electron within a band due to electron–phonon scattering does not modify its energy much, in contrast to the situation for the more curved bands of PbS and PbSe. As a result, cooling could be slower in MoTe_2_ and compete less effectively with CM than in the case for PbS and PbSe.

The best fitted values of *F*_CM_/*R*_cool_ are 349 ± 315 eV for MoTe_2_, 766 ± 393 eV for PbS, and 83.9 ± 28.7 eV for PbSe. The relatively large uncertainty of *F*_CM_/*R*_cool_ of MoTe_2_ can be attributed to its highly increasing *N̅*_CM_ as a function of photon energy. As can be seen in Figures S2 and S3, the values of *N̅*_CM_ for MoTe_2_ increase so rapidly with photon energy that adjusting *F*_CM_/*R*_cool_ from 1 eV to 10^6^ eV has a much smaller impact on the quantum yield than is the case for PbS and PbSe. This therefore leads to a larger uncertainty in the parameter *F*_CM_/*R*_cool_ for MoTe_2_. Moreover, Figure S2 also shows that the CM efficiency in MoTe_2_ can predominately be attributed to its large and rapidly increasing number of CM pathways, as large changes in *F*_CM_/*R*_cool_ only slightly affect the quantum yield.

The large uncertainty of *F*_CM_/*R*_cool_ in the materials indicates that quantitative comparison must be done with caution. The large uncertainty of the value of *F*_CM_/*R*_cool_ for MoTe_2_ makes it unmeaningful to compare it with those of PbS and PbSe. However, the *F*_CM_/*R*_cool_ values of PbS and PbSe do show a significant difference that cannot be attributed solely to the fitting uncertainty and therefore requires further discussion. The value of *F*_CM_/*R*_cool_ of PbSe is smaller than that of PbS because the value of *N̅*_CM_ is larger in PbSe than in PbS at any given photon energy, whereas the quantum yield of both materials is similar. This is illustrated in Figure S3a,b. Note that the difference between Figures S3 and 3 is the horizontal axis, which is defined as photon energy in eV in Figure S3 rather than band gap multiple *ℏ*ω/*E*_g_ in Figure 3. Since the value of *N̅*_CM_ of PbSe is greater than that of PbS while the quantum yields are similar, the *F*_CM_/*R*_cool_ value of PbSe must be lower to compensate for its larger *N̅*_CM_ value. This implies that PbSe exhibits a larger *R*_cool_ and/or a smaller *F*_CM_ than PbS. *F*_CM_ is inversely proportional to the square of the dielectric function, i.e., .^33,34^ The dielectric constants of PbS and PbSe are 169 and 210, respectively.^54^ We therefore estimate the *F*_CM_ value of PbS to be roughly 1.5 times larger than that of PbSe. Details of the physical meaning and unit of *F*_CM_ are discussed in the Supporting Information. Stewart et al. estimated that the energy-loss rate due to Fröhlich-type electron–phonon coupling is two times larger in PbS than in PbSe, due to a higher longitudinal optical phonon energy and stronger electron–phonon polar coupling.^55^ The cooling rate *R*_cool_ is therefore expected to be twice as large in PbS. The combined estimates of the relative values of *F*_CM_ and *R*_cool_ predict that the *F*_CM_/*R*_cool_ of PbS is 1.5/2.0 = 0.75 times that of PbSe, which is clearly in disagreement with our findings.

The cause of the discrepancy between the relative values of *F*_CM_/*R*_cool_ of PbS and PbSe obtained by fitting and the above-mentioned estimation is unclear. As shown in Figure 2, PbS and PbSe have a similar band structure with the main difference being that PbSe has a band gap of 0.26 eV and PbS a band gap of 0.42 eV.^51^ The difference in band gap will play a minor role, since the actual CM onset for both is at much higher energy near 2 eV; see Figure S2. Understanding the different fitted values of *F*_CM_/*R*_cool_ for PbS and PbSe asks for calculating *F*_CM_, which involves calculating the Coulomb matrix elements with the initial and final state wave functions, and measuring or calculating *R*_cool_. This is, however, out of the scope of this work.

From the fitted CM probability, we can back calculate the effective ratio of the CM and cooling rates for electrons and holes separately, with the following expression
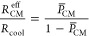
15where , with *P*_CM_^*i*^ describing the probability for CM of an electron in state *i* = *e*_*i*_, or a hole in state *i* = *h*_*i*_, see eqs 6 and 7. Note that *P*_CM_^*i*^ depends on the density of CM pathways and this will be reflected in the effective ratio *R*_CM_^eff^/*R*_cool_. Figure 4d shows the effective *R*_CM_^eff^/*R*_cool_ for electrons, holes, and the average *R*_CM_^eff^/*R*_cool_ of both carriers as a function of band gap multiple. It can be seen that the slope of *R*_CM_^eff^/*R*_cool_ is steepest for MoTe_2_, and then, it is steepest for PbS and PbSe, respectively. Equation 15 shows that when the ratio *R*_CM_^eff^/*R*_cool_ for electrons and holes each reaches unity, *P̅*_CM_ = 0.5 for both, and so the total probability to generate a secondary electron–hole pair is 1, resulting in a total quantum yield of 2. As can be seen from comparing Figure 4a–d, this point is reached at a photon energy of 3.5*E*_g_ (3.0 eV) for MoTe_2_, while this occurs at a much higher energy of 8.3*E*_g_ (3.5 eV) for PbS and at 13.8*E*_g_ (3.6 eV) for PbSe. This variation of *R*_CM_^eff^/*R*_cool_ with *ℏ*ω/*E*_g_ in Figure 4d reflects the trends of the experimental quantum yields in Figure 4a–c. The high CM quantum yield as a function of *ℏ*ω/*E*_g_ for MoTe_2_ as compared to PbS and PbSe is thus due to the relatively high density of CM pathways (see Figure 3a), yielding a high CM rate that effectively outcompetes relatively slow cooling.

In addition, Figure 4d shows that *R*_CM_^eff^/*R*_cool_ for MoTe_2_ above 3.2*E*_g_ (2.7 eV) is larger for holes than for electrons. This is caused by a difference in the distribution of the density of the CM pathways, *N*_CM_, of the initial electron and hole states. The distribution of *N*_CM_ for all photogenerated initial states (i.e., all possible combinations of initial electron and hole states at a certain photon energy) is shown in Figure S4. From the upper panel for MoTe_2_, it can be seen that while the mean value of *N*_CM_ is typically similar for electrons and holes, above 3 eV the *N*_CM_ distribution for holes becomes narrower, and the median becomes higher than that of the electrons. This means that at photon energies above 3 eV, a larger fraction of photogenerated holes have a high number of CM pathways than is the case for electrons. This makes CM decay more likely for holes than for electrons above 3 eV. For PbS and PbSe, the *N*_CM_ distribution is similar for electrons and holes, which leads to comparable CM rates for both carriers.

According to the theoretical analysis above, the high CM efficiency of MoTe_2_ is mainly due to an extraordinarily high density of CM pathways that goes paired with a beneficial relatively slow cooling rate. These effects can be attributed to the band structure diagram of MoTe_2_ (Figure 2), which shows a large density of relatively flat bands as opposed to the case of PbS and PbSe. The flatness of the bands of MoTe_2_ reduces cooling, and their large density results in many CM pathways with conservation of electronic energy and crystal momentum. By contrast, the bands in PbS and PbSe are more curved leading to faster intraband cooling of electrons and holes and the smaller density of states leads to less CM pathways.

## Conclusions

We calculated the density of CM decay pathways, *N*_CM_, for the bulk materials 2H–MoTe_2_, PbS, and PbSe, using electronic band structures from DFT calculations. The variation of *N*_CM_ with photon energy averaged for electrons and holes, already qualitatively describes the much more efficient CM in 2H–MoTe_2_, as compared to PbS and PbSe. We take into account cooling of charge carriers in competition with CM, by introducing a fit parameter. In this way, our model quantitatively reproduces the disparate experimental CM quantum yields for the above-mentioned materials in a quantitative manner. The relatively small changes induced by adjusting the fit parameter confirms that the high CM efficiency of 2H–MoTe_2_ can be mainly attributed to its high density of CM pathways. Our calculations predict a higher probability of CM for holes than for electrons in MoTe_2_ at photon energies above 3 eV, while these are found to be similar for PbS and PbSe. The theoretical model can be applied to screen new materials for the prospects of efficient CM.

## Supporting Information Available

The Supporting Information is available free of charge at https://pubs.acs.org/doi/10.1021/acs.jpcc.4c00383. The code that was used for the calculation of *N*_CM_, *cmscript.py*, is available upon reasonable request

Derivation of eq 8 with definition of the prefactor *N*_CM_, discussion of convergence of density of CM decay pathways with the value of energy tolerance ε_tol_ and the number of *k*-points, and box plots showing distributions of density of electron and hole decay pathways as a function of energy (PDF)
