# Supplementary material for: New Theoretical Model to Describe Carrier Multiplication in Semiconductors: Explanation of Disparate Efficiency in MoTe2 versus PbS and PbSe
Source: J Phys Chem C Nanomater Interfaces. 2024 Feb 28;128(9):3693–702. doi: 10.1021/acs.jpcc.4c00383 (PMC10926152; doi:10.1021/acs.jpcc.4c00383)
Supplement: Supplementary file 1 — jp4c00383_si_001.pdf [file jp4c00383_si_001.pdf]

SUPPORTING INFORMATION

New Theoretical Model to Describe Carrier Multiplication in Semiconductors: Explanation of Disparate Efficiency in $MoTe_2$ versus PbS and PbSe

Sven Weerdenburg,[1] Nisha Singh,[1] Marco van der Laan,[3] Sachin Kinge,[2] Peter Schall,[3] and Laurens D. A. Siebbeles[1,*]

1. Chemical Engineering Department, Delft University of Technology, Van der Maasweg 9, 2629 HZ Delft, The Netherlands

2. Materials Research & Development, Toyota Motor Europe, B1930 Zaventem, Belgium

3. Institute of Physics, University of Amsterdam, 1098 XH Amsterdam, The Netherlands

*Corresponding author email address: L.D.A.Siebbeles@tudelft.nl

1. Crystal structures

Table S1. Crystal structure data used in DFT calculations.

Material	Lattice, space group	Primitive lattice vectors	Lattice constants (Å)	Atom positions		Reference
2H-$MoTe_2$	Hexagonal, $P6_3/mmc$ [194]	$\boldsymbol{a_1} = \left(\frac{a}{2}, -\frac{a\sqrt{3}}{2}, 0\right)$ $\boldsymbol{a_2} = \left(\frac{a}{2}, \frac{a\sqrt{3}}{2}, 0\right)$ $\boldsymbol{a_3} = (0, 0, c)$	a = 3.519 c = 13.964	Mo:	$\pm(\frac{1}{3}, \frac{2}{3}, \frac{1}{4})$	[1]
				Te:	$\pm(\frac{1}{3}, \frac{2}{3}, z)$ $\pm(\frac{2}{3}, \frac{1}{3}, z + \frac{1}{2})$ z = 0.625	
PbS	FCC, Fm$\overline{3}$m [225]	$\boldsymbol{a_1} = \left(0, \frac{a}{2}, \frac{a}{2}\right)$ $\boldsymbol{a_2} = \left(\frac{a}{2}, 0, \frac{a}{2}\right)$ $\boldsymbol{a_3} = \left(\frac{a}{2}, \frac{a}{2}, 0\right)$	a = 5.94	Pb:	(0,0,0)	[2]
				S:	$(\frac{1}{2}, \frac{1}{2}, \frac{1}{2})$	
PbSe	FCC, Fm$\overline{3}$m [225]	$\boldsymbol{a_1} = \left(0, \frac{a}{2}, \frac{a}{2}\right)$ $\boldsymbol{a_2} = \left(\frac{a}{2}, 0, \frac{a}{2}\right)$ $\boldsymbol{a_3} = \left(\frac{a}{2}, \frac{a}{2}, 0\right)$	a = 6.12	Pb:	(0,0,0)	[2]
				Se:	$(\frac{1}{2}, \frac{1}{2}, \frac{1}{2})$	

2. Derivation of the carrier multiplication rate

The carrier multiplication (CM) rate, also known as the impact ionization rate, is calculated from Fermi's Golden Rule.[3–9] The rate for a carrier decaying from the state with index 1 to state 1', while exciting an electron from state 2 to 2' is then

$$r_{CM}(1,2;1',2') = \frac{2\pi}{\hbar} T(1,2;1',2') \delta(\Delta E_{1',2',1,2}) \tag{S1}$$

where $\delta(\Delta E_{1',2',1,2})$ is the Dirac delta function with the energy difference between final and initial states given by $\Delta E_{1',2',1,2} = E_{1'} + E_{2'} - E_1 - E_2$. The factor $T(1,2;1',2')$ consists of the direct and exchange matrix elements of the Coulomb operator involving the initial and final electronic states.[10,11] Each state in eq S1 and below is defined by a crystal momentum and band index. For instance, the label 1 is implies that the crystal momentum is $\boldsymbol{k}_1$ and the band index is ν_1.

To calculate the total CM rate, $R_{CM}(1)$, for a primary electron in state 1, the rates for all possible CM decay pathways need to be calculated and summed, i.e., $R_{CM}^1 = \sum_2 \sum_{1'} \sum_{2'} r_{CM}(1,2;1',2')$. As discussed in the main text, we replace the Dirac delta function in eq S1 by a top-hat function divided by $2\varepsilon_{tol}$, and we introduce the function $I(E_1,E_{1'},E_2,E_{2'})$ defined in eq 4 in the main text to fulfill energy conservation requirements of CM. This then yields the total CM rate

$$R_{CM}^1 = \frac{2\pi}{\hbar}\sum_{\mathbf{k}_2}\sum_{\mathbf{k}_{1'}}\sum_{\mathbf{k}_{2'}}\sum_{\nu_2}\sum_{\nu_{1'}}\sum_{\nu_{2'}} T(1,2;1',2')\delta_{\mathbf{G},\,\mathbf{k}_{1'}+\mathbf{k}_{2'}-\mathbf{k}_1-\mathbf{k}_2}\frac{\Theta(\Delta E_{1',2',1,2},\epsilon_{tol})}{2\varepsilon_{tol}} I(E_1,E_{1'},E_2,E_{2'}) \quad \text{(S2)}$$

where the Kronecker delta function ensures conservation of crystal momentum. Following previous studies[6,8,9,12–18] we neglect the dependence of the Coulomb matrix elements on the initial and final states. We can then define $T(1,2;1',2') \equiv T_{CM}$ and take this factor out of the summation in eq S2. Using the definition of N_{CM}^1 in eq 2 of the main text then allows us to write the CM rate as

$$R_{CM}^1 = \frac{2\pi}{\hbar}T_{CM}N_{CM}^1 \equiv F_{CM}N_{CM}^1 \quad \text{(S3)}$$

with the prefactor $F_{CM} = \frac{2\pi}{\hbar}T_{CM}{n_k}^2$. As discussed in the main text below eq 2, the value of N_{CM}^1 corresponds to the fraction of all ${n_k}^2$ final states 1' and 2' per unit energy that can be reached by CM for each combination of band indices υ_i. F_{CM} is thus proportional to the hypothetical CM rate for one combination of band indices υ_i without the constraints of conservation of crystal momentum and energy, i.e., all ${n_k}^2$ pathways are included. The proportionality constant between F_{CM} and the hypothetical rate is the inverse of the energy unit of the tolerance interval for energy conservation ($2\varepsilon_{tol}$), which is electronvolt in our work. Hence, the unit of F_{CM} is eV/s and the unit of F_{CM}/R_{cool} is eV. For a carrier in the initial state 1 the ratio of the CM and cooling rates is $F_{CM}N_{CM}^1/R_{cool}$. This ratio is indeed dimensionless, since the dimension of N_{CM}^1

is eV^{-1} due to the presence of the energy normalized top-hat function $\Theta(\Delta E,\varepsilon_{tol})/2\varepsilon_{tol}$, see eq (2) in the main text. Note that an increase in n_k implies an increase of the volume, since $V = n_k$ V_{uc}, where V_{uc} is the volume of the unit cell. Since N_{CM}^1 is normalized to ${n_k}^2$ only, its dimension does not contain a volume factor.

As discussed in the main text, the top-hat function $\Theta(\Delta E,\varepsilon_{tol})$ is used to relax energy conservation. As a consequence, the number of CM pathways that satisfy the relaxed energy conservation rule, will scale with the value that is chosen for ε_{tol}. If it is assumed that the density of final states does not change significantly within an interval of $(-\varepsilon_{tol},\varepsilon_{tol})$, i.e. $\rho(E_f)$ $= \rho_f$ for $-\varepsilon_{tol} \leq E_f \leq \varepsilon_{tol}$, where ρ_f is a constant, then the number of final states is calculated from $n_f = \int_{-\varepsilon_{tol}}^{\varepsilon_{tol}} \rho_f d\mathrm{E_f} = 2\varepsilon_{tol}\rho_f$. The number of final states thus scales with a factor of $2\varepsilon_{tol}$ when $\Theta(\Delta E,\varepsilon_{tol})$ is introduced. To correct for this, the normalization factor $\frac{1}{2\varepsilon_{tol}}$ is included in eq 2 of the main text. To verify that the value of ε_{tol} was sufficiently small so that ρ_f can be considered constant, we calculated $\overline{N}_{CM}$ for PbS, using 30 meV and 60 meV as values for ε_{tol}. As can be seen from Figure S1 and Table S1, the prefactor $\frac{1}{2\varepsilon_{tol}}$ indeed ensures that N_{CM} does not depend on ε_{tol}.

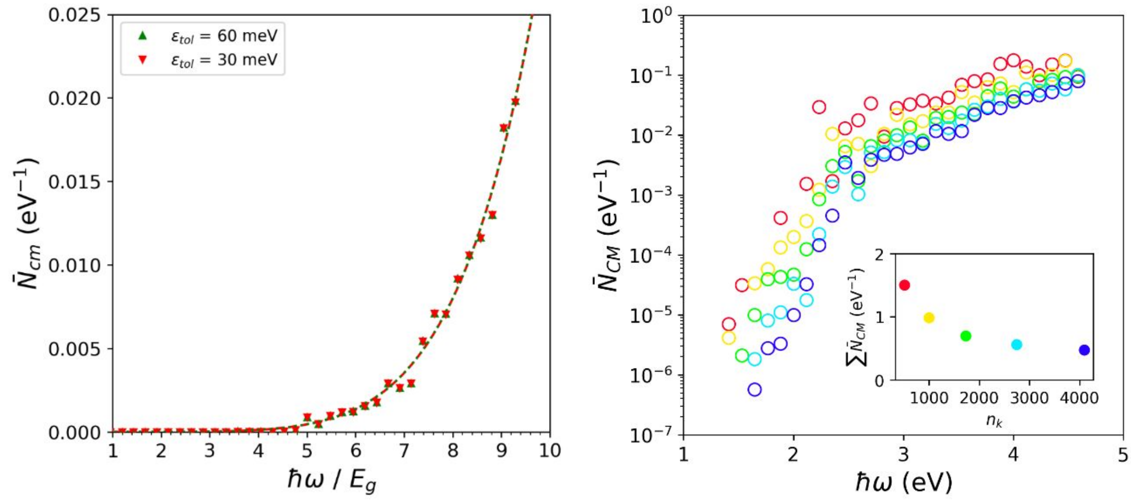

Figure S1. Left panel: $\overline{N}_{CM}$ calculated for PbS, using values 30 meV and 60 meV for ε_{tol}, and the fitted cures with the fitting parameters given in Table S2. Right panel: $\overline{N}_{CM}$ calculated for PbS from *k*-point grids: 8x8x8 (red), 10x10x10 (yellow), 12x12x12 (green), 14x14x14 (cyan), 16x16x16 (blue).

Table S2. The fitting parameters obtained from fitting eq 11 of the main text to the calculated $\overline{N}_{CM}$ data for PbS in Figure S1.

Material	*A* (eV^{-1})	*P*
PbS ($\varepsilon_{tol} =$ 30 meV)	$2.3429 \cdot 10^{-8}$	6.126
PbS ($\varepsilon_{tol} =$ 60 meV)	$2.3420 \cdot 10^{-8}$	6.128

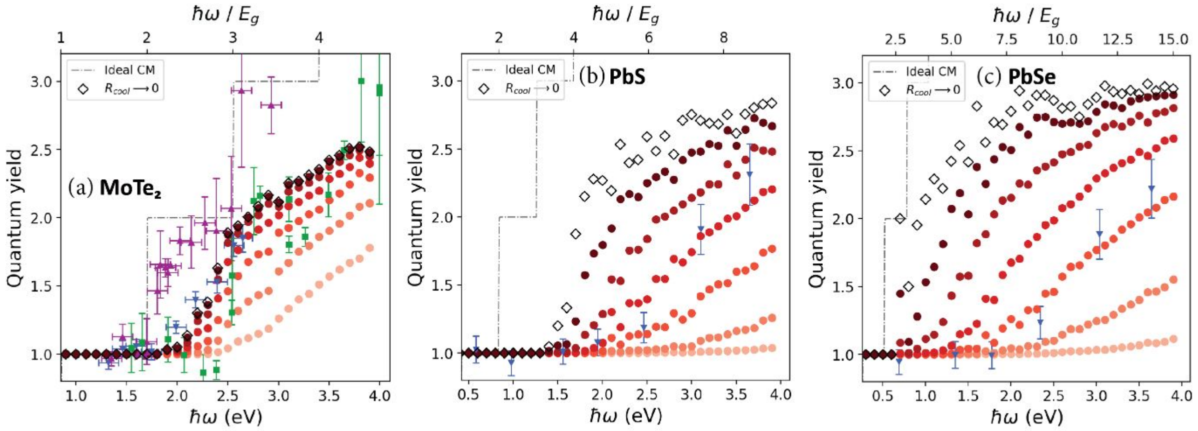

Figure S2 – Experimental and calculated quantum yield with varying F_{CM}/R_{cool} from 1 to 10^6 for (a) $MoTe_2$[19,20], (b) PbS[21], and (c) PbSe[21]. The darkness of the dots representing the calculated quantum yields increases as the value of F_{CM}/R_{cool} goes up. For $MoTe_2$ the PIA,[19] PIB[19] and THz[20] shown as purple, blue, and green symbols, respectively. For PbS and PbSe, the THz measurements[21] are shown as blue symbols.

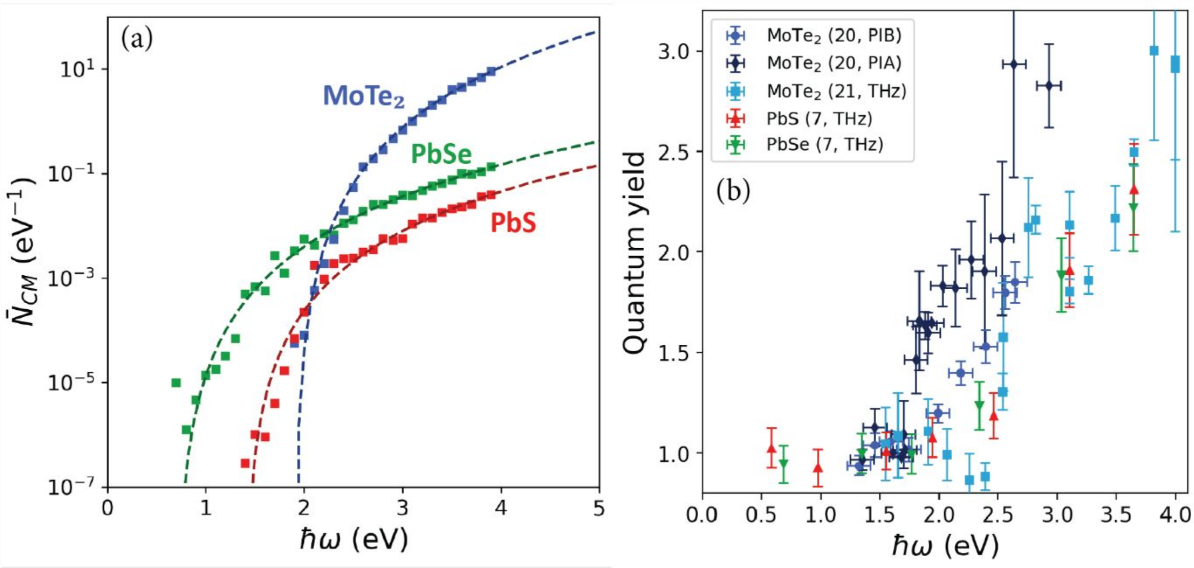

Figure S3 – (a) Calculated $\overline{N}_{CM}$ as function of energy on logarithmic scale. (b) Experimental quantum yield as function of energy for $MoTe_2$[19,20], PbS[21], and PbSe[21].

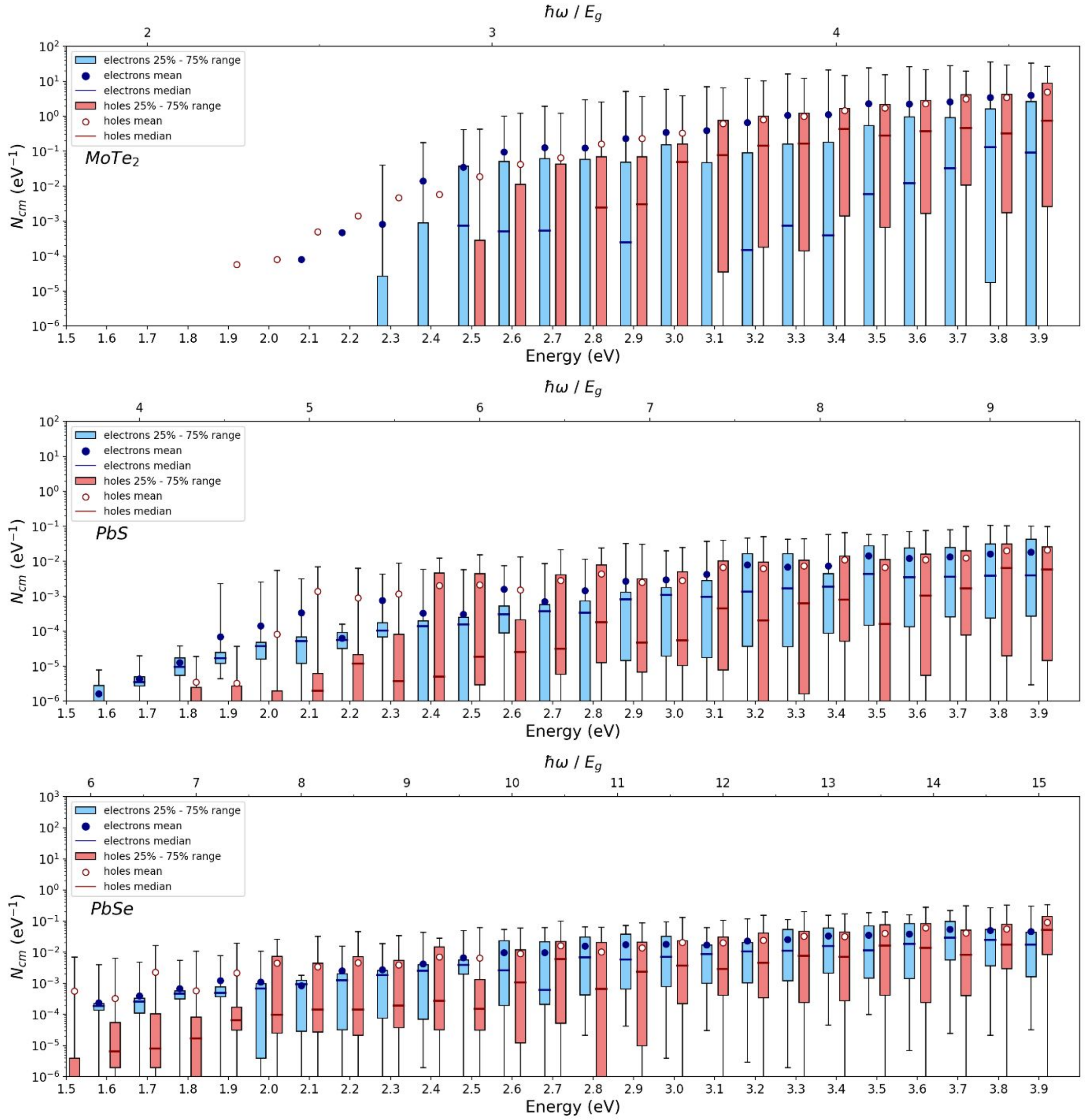

Figure S4. Box plots showing the distribution of N_{CM} for electrons and holes for $MoTe_2$, PbS and PbSe on energy intervals of 0.1 eV. The colored boxes depict the range between the first to the third quartile of the calculated N_{CM} distribution for the electrons (blue) and holes (red). The circles depict the mean for the electrons (filled) and holes (empty) and the horizontal lines indicates the median. The whiskers extend from the minimum to the maximum values of N_{CM}

REFERENCES

(1) Puotinen, D.; Newnham, R. E. The Crystal Structure of MoTe 2. *Acta Crystallogr.* **1961**, *14* (6), 691–692.

(2) Khokhlov, D. *Lead Chalcogenides Physics and Applications*; OPTOELECTRONIC PROPERTIES of SEMICONDUCTORS and SUPERLATTICES; Taylor & Francis, 1970; Vol. 18.

(3) Stobbe, M.; Könies, A.; Redmer, R.; Henk, J.; Schattke, W. Interband Transition Rate in GaAs. *Phys. Rev. B* **1991**, *44* (20), 11105–11110.

(4) Devreese, J. T.; van Welzenis, R. G.; Evrard, R. P. Impact Ionisation Probability in InSb. *Appl. Phys. Solids Surf.* **1982**, *29* (3), 125–132.

(5) Kamakura, Y.; Mizuno, H.; Yamaji, M.; Morifuji, M.; Taniguchi, K.; Hamaguchi, C.; Kunikiyo, T.; Takenaka, M. Impact Ionization Model for Full Band Monte Carlo Simulation. *J. Appl. Phys.* **1994**, *75* (7), 3500–3506.

(6) Sano, N.; Yoshii, A. Impact-Ionization Model Consistent with the Band Structure of Semiconductors. *J. Appl. Phys.* **1995**, *77* (5), 2020–2025.

(7) Harrison, D.; Abram, R. A.; Brand, S. Impact Ionization Rate Calculations in Wide Band Gap Semiconductors. *J. Appl. Phys.* **1999**, *85* (12), 8178–8185.

(8) Harrison, D.; Abram, R. A.; Brand, S. Characteristics of Impact Ionization Rates in Direct and Indirect Gap Semiconductors. *J. Appl. Phys.* **1999**, *85* (12), 8186–8192.

(9) Kane, E. O. Electron Scattering by Pair Production in Silicon. *Phys. Rev.* **1967**, *159* (3), 624–631.

(10) Sano, N.; Yoshii, A. Impact-Ionization Theory Consistent with a Realistic Band Structure of Silicon. *Phys. Rev. B* **1992**, *45* (8), 4171–4180.

(11) Stobbe, M.; Redmer, R.; Schattke, W. Impact Ionization Rate in GaAs. *Phys. Rev. B* **1994**, *49* (7), 4494–4500.

(12) Fischetti, M. V.; Sano, N.; Laux, S. E.; Natori, K. Full-Band Monte Carlo Simulation of High-Energy Transport and Impact Ionization of Electrons and Holes in Ge, Si, and GaAs. In *1996 International Conference on Simulation of Semiconductor Processes and Devices. SISPAD '96 (IEEE Cat. No.96TH8095)*; Japan Soc. Appl. Phys: Tokyo, Japan, 1996; pp 43–44.

(13) Dinh, T. V.; Jungemann, C. Impact Ionization Rates for Strained Si and SiGe. *Solid-State Electron.* **2009**, *53* (12), 1318–1324.

(14) Lansdsberg, P. T.; Robbins, D. J. The First 70 Semiconductor Auger Processes. *Solid-State Electrons* **1978**, *21* (11–12), 1289–1294.

(15) Franceschetti, A.; An, J. M.; Zunger, A. Impact Ionization Can Explain Carrier Multiplication in PbSe Quantum Dots. *Nano Lett.* **2006**, *6* (10), 2191–2195.

(16) Allan, G.; Delerue, C. Influence of Electronic Structure and Multiexciton Spectral Density on Multiple-Exciton Generation in Semiconductor Nanocrystals: Tight-Binding Calculations. *Phys. Rev. B* **2008**, *77* (12), 125340.

(17) Delerue, C.; Allan, G.; Pijpers, J. J. H.; Bonn, M. Carrier Multiplication in Bulk and Nanocrystalline Semiconductors: Mechanism, Efficiency, and Interest for Solar Cells. *Phys. Rev. B* **2010**, *81* (12), 125306.

(18) Rabani, E.; Baer, R. Distribution of Multiexciton Generation Rates in CdSe and InAs Nanocrystals. *Nano Lett.* **2008**, *8* (12), 4488–4492.

(19) Kim, J. H.; Bergren, M. R.; Park, J. C.; Adhikari, S.; Lorke, M.; Frauenheim, T.; Choe, D. H.; Kim, B.; Choi, H.; Gregorkiewicz, T.; Lee, Y. H. Carrier Multiplication in van Der Waals Layered Transition Metal Dichalcogenides. *Nat. Commun.* **2019**, *10* (1), 1–9.

(20) Zheng, W.; Bonn, M.; Wang, H. I. Photoconductivity Multiplication in Semiconducting Few-Layer MoTe2. *Nano Lett.* **2020**, *20* (8), 5807–5813.

(21) Pijpers, J. J. H.; Ulbricht, R.; Tielrooij, K. J.; Osherov, A.; Golan, Y.; Delerue, C.; Allan, G.; Bonn, M. Assessment of Carrier-Multiplication Efficiency in Bulk PbSe and PbS. *Nat. Phys.* **2009**, *5* (11), 811–814.
